# The role of gut mycobiome in responses to cancer immunotherapy

**DOI:** 10.1080/19490976.2025.2571433

**Published:** 2025-10-29

**Authors:** Natalia Szóstak, Piotr Kozłowski, Tao Zuo, Anna Philips

**Affiliations:** a Institute of Bioorganic Chemistry, Polish Academy of Sciences, Poznań, Poland; b Guangdong Institute of Gastroenterology, The Sixth Affiliated Hospital, Sun Yat-sen University, Guangzhou, China; c Key Laboratory of Human Microbiome and Chronic Diseases (SunYat-sen University), Ministry of Education, Guangzhou, China

**Keywords:** Gut mycobiota, gut fungi, mycobiome, gut dysbiosis, cancer, immunotherapy, ICI, ICB

## Abstract

The gut microbiome has emerged as an important modulator of cancer progression and therapy response, yet the role of its fungal component, the gut mycobiome, remains poorly understood. In this review, we summarize current knowledge on the relationship between intestinal fungi and cancer, with a particular focus on the potential influence of the gut mycobiome on the efficacy of immune checkpoint inhibitors (ICIs). Drawing from both human studies and preclinical models, we discuss evidence linking fungal dysbiosis to altered immune responses and therapy outcomes in cancer patients. Specific fungal taxa, such as *Candida* and *Malassezia*, have been associated with changes in the tumor microenvironment and modulation of host immunity. We also review the proposed mechanisms through which fungi may impact antitumor immunity, including interactions with immune receptors and production of bioactive metabolites. Although research in this field is still in its early stages, emerging data suggest that the gut mycobiome may serve as a biomarker for immunotherapy response and a potential target for therapeutic intervention. A better understanding of host–fungus interactions in the gut could contribute to the development of personalized strategies to improve the effectiveness and safety of cancer immunotherapy.

## Introduction

Immunotherapy has emerged as a transformative strategy in the treatment of cancer, fundamentally altering therapeutic landscapes and patient outcomes. The development and clinical deployment of immune checkpoint inhibitors (ICIs), particularly those targeting programmed death-1 receptor (PD-1), its ligand PD-L1, and cytotoxic T-lymphocyte antigen-4 (CTLA-4), have yielded unprecedented responses in a range of malignancies including but not limited to melanoma,^1^^,^^2^ non-small cell lung cancer (NSCLC),^3^^,^^4^ and renal cell carcinoma (RCC).^5^^,^^6^ In parallel, advances in adoptive cell therapy, such as chimeric antigen receptor (CAR)-T cells,^7^ and cytokine-based treatments^8^ have further expanded the arsenal of immuno-oncological tools.

Despite these advances, many patients do not respond to ICIs^9^ or develop immune-related adverse events (irAEs),^10^ prompting efforts to identify factors that influence treatment outcomes. These challenges have led to a growing body of research into the factors influencing immunotherapy efficacy, with the gut microbiota emerging as a critical modulator of treatment outcomes. Landmark studies have demonstrated that specific bacterial taxa within the gut can affect the activity of ICIs, modulate systemic cytokine profiles, and enhance tumor-specific immune responses.^11-15^ For example, *Candida albicans* overgrowth has been linked to colorectal and liver cancer progression, while *Malassezia* expansion contributes to pancreatic carcinogenesis through complement activation.^16-20^

While the bacteriome has been extensively characterized in this context, considerably less attention has been paid to the fungal component of the gut microbiota – the mycobiome. This underexplored microbial kingdom comprises a smaller, yet functionally potent, proportion of the gastrointestinal ecosystem. The mycobiome includes commensal, opportunistic, and transient fungal species belonging to *Candida*, *Malassezia*, *Saccharomyces*, and *Cladosporium* genera, among others, which interact with host immunity and microbial communities within a human body.^21-24^ Historically, the study of gut fungi has been hampered by technical challenges, including lower fungal biomass, difficulty in DNA extraction, and limited reference databases.^25^ However, recent advances in sequencing technologies and bioinformatics have begun to overcome these barriers, enabling more accurate profiling of the gut mycobiome and revealing its potential as an independent or synergistic modulator of immune responses.

Although fungi constitute only a minor fraction of the gut microbiome, they possess unique cellular components, including ergosterol-containing membranes, chitin-based cell walls, and large genomes capable of encoding a broad array of virulence factors. Fungal pathogen-associated molecular patterns (PAMPs) such as *β*-glucans, mannans, and chitin are recognized by innate immune receptors, particularly C-type lectin receptors (CLRs),^26^^,^^27^ the main class of fungal-sensing pattern recognition receptors, including Dectin-1, Toll-like receptor 2 (TLR2), and Mincle. Their engagement triggers inflammatory signaling cascades and modulates adaptive immunity, influencing T cell development and polarization, dendritic cells (DCs) function, and the production of key cytokines such as interleukin (IL)-17 and IL-22, central to mucosal immune responses.^28^^,^^29^

Furthermore, fungi contribute to the gut metabolome by producing a variety of substances, including ethanol, acetaldehyde, oxylipins, and other bioactive lipids that may modulate epithelial integrity, immune tolerance, and cross-talk with bacterial symbionts.^30-32^ When the balance of the mycobiome is disrupted, such as through overgrowth of particular species or loss of fungal diversity, these metabolic functions can shift toward proinflammatory or pathogenic outcomes. Such dysbiosis has been linked to gastrointestinal and systemic inflammatory diseases, and emerging evidence suggests it may also play a role in cancer development and treatment response.

In this review, we provide emerging evidence on the role of the gut mycobiome in cancer immunotherapy. We discuss its composition, functional capabilities, and interactions with the host immune system. We also explore its impact on tumor progression and immunotherapy efficacy.

## The gut mycobiome in cancer

Fungal dysbiosis in cancer is not limited to changes in abundance or diversity; it also entails profound shifts in functional capacity, with direct consequences for host immunity and metabolism. In cancer patients, the gut mycobiome undergoes substantial remodeling, both in taxonomic composition and ecological structure. These alterations are increasingly recognized as important modulators of disease progression, immune dynamics, and responsiveness to therapy. A key aspect of this remodeling is the change in fungal diversity. For instance, colorectal cancer (CRC) and hepatocellular carcinoma (HCC) are both characterized by a markedly reduced richness of gut fungal communities.^18-20^ However, this pattern is not universal: fungal diversity may increase in certain extraintestinal tumors such as lung adenocarcinoma and melanoma, likely reflecting differences in the local conditions rather than gut-specific effects.^16^^,^^17^ These differences likely reflect distinct local conditions, such as tissue oxygenation, nutrient availability, immune surveillance, and epithelial barrier properties.

Regardless of directionality, fungal dysbiosis in cancer is typified by reproducible taxonomic shifts. Although the fungal composition varies by cancer type, specific signatures have been reported: *Candida* spp. enrichment in CRC and HCC, *Malassezia* expansion in pancreatic cancer (PDA), *Aspergillus* in CRC, and increased diversity with enrichment of *Saccharomyces* and *Aspergillus* in lung adenocarcinoma and melanoma.^16-20^ Overall, opportunistic and immunomodulatory genera, especially *Candida*, *Malassezia*, or *Aspergillus*, are frequently enriched across tumor types. Among them, *Candida tropicalis* and *C. albicans* have emerged as key contributors to the altered fungal landscape, with consistent overrepresentation reported in CRC, HCC, PDA and lung, and skin cancers.^33-40^ Both species contribute to disease pathology via secretion of secreted aspartyl proteases (SAPs), phospholipases, and candidalysin, which disrupt epithelial barriers, activate Th17 responses, and promote chronic inflammation, thereby reshaping the tumor microenvironment.^41^

In CRC, a significant increase in fungal burden, especially of *C. tropicalis*, is accompanied by a notable reduction in overall fungal diversity compared to healthy individuals.^34-36^ Furthermore, the *Ascomycota*/*Basidiomycota* ratio, a marker of ecological imbalance within the fungal community, is increased in CRC patients.^42^ Taxa such as *Candida*, *Malassezia*, and *Aspergillus flavus* (a known aflatoxin producer) are consistently enriched in this context.^35^^,^^42^ *Candida* spp. contribute to disease pathology through the production of virulence factors such as SAPs, phospholipases, and candidalysin, which collectively damage epithelial barriers and trigger pro-inflammatory cytokine responses.^41^

In HCC, intestinal overgrowth of *C. albicans*, often alongside *Malassezia furfur*, has been associated with microbial imbalance and enhanced gut inflammation.^37^ These alterations may influence both local mucosal immunity and systemic immune responses.

In PDA, gut enrichment of *Malassezia* is linked to complement activation via the mannose-binding lectin (MBL) pathway and to enhanced IL-33 secretion, both of which contribute to a pro-inflammatory milieu.^43^ Moreover, *C. albicans* plays a central role in shaping antifungal T helper (Th)17 immunity in humans, acting as the dominant antigen for Th17 cell activation. Intestinal inflammation can drive the expansion of both *C. albicans*-specific and cross-reactive Th17 cells, potentially impacting immune surveillance and tumor progression.^38^

Remarkably, the influence of the gut mycobiome extends beyond gastrointestinal malignancies to anatomically distant cancers, such as those of the lung and bladder. In lung adenocarcinoma, for example, fungal diversity is paradoxically increased. This shift is characterized by the enrichment of genera such as *Saccharomyces*, *Aspergillus*, and *Apiotrichum*, while *Candida* species appear to be diminished.^44^ These findings suggest that distinct fungal signatures may be linked to tumor-specific immune landscapes. For example, in melanoma, enrichment of *C. albicans* and *Malassezia restricta* was associated with increased PD-L2 expression and regulatory immune cell infiltration,^39^^,^^45^ while in CRC, fungal dysbiosis drives Th17 polarization and MDSC accumulation,^34^^,^^41^ both shaping tumor-specific immune landscapes.

In our study,^39^ we found that gut mycobiota composition differed significantly between melanoma patients and healthy individuals and underwent dynamic changes during anti-PD-1 immunotherapy. Melanoma patients exhibited an increased abundance of potentially pro-inflammatory fungi such as *C. albicans*, *Candida dubliniensis*, *Neurospora crassa*, and *M. restricta*, and decreased levels of potentially beneficial species like *Saccharomyces cerevisiae* and *Debaryomyces hansenii*. Notably, *C. albicans* and *M. restricta* levels increased during immunotherapy and were associated with poorer response to the therapy and overall worse clinical outcomes, including higher risk of disease progression and shorter progression-free survival. Conversely, *Saccharomyces paradoxus* was associated with treatment response. These changes correlated with immune profiles: responders showed lower fungal diversity and positive correlations between *C. albicans* and markers of adaptive immunity, while in non-responders, *C. albicans* correlated with regulatory and suppressive immune cells. Of note, *M. restricta* was linked to increased PD-L2 expression in non-responders, potentially contributing to immune evasion.^45^

Crucially, the gut mycobiome does not operate in isolation. Emerging evidence suggests that the mycobiome interacts with the bacteriome and virome to shape the overall immunological tone of the gut and distant tissues.^46-48^ Interkingdom interactions between fungi and bacteria, encompassing both competition and metabolic interplay, can significantly influence host immunity. For instance, *Candida* overgrowth in the gut has been associated with increased lactate-producing bacteria and a concurrent decrease in short-chain fatty acid (SCFA)-producing taxa. Recent findings by Seelbinder et al. demonstrated that in lung cancer patients, expansion of intestinal *Candida* was driven by an ecological shift characterized by increased lactate-producing bacteria and reduced SCFA producers. This niche favored fungal proliferation by enabling *Candida* to utilize lactate as a nutrient, giving it a competitive advantage over other fungi.^49^ This microbial shift alters the mucosal immune environment and may have downstream effects on systemic anti-tumor immune responses.^17^^,^^50^ Co-aggregation of fungi and bacteria in biofilms, as well as metabolic exchange between them, further exacerbate inflammation and immune evasion.^51^^,^^52^

These observations point to a dynamic role of the gut mycobiome in modulating host immunity during cancer. Depending on the fungal species present, their abundance, and their interactions with immune cells, the gut mycobiome can either promote tumor-associated inflammation or support anti-tumor immune surveillance, reflecting its dual and context-dependent role in cancer. Through systemic cytokine modulation, epithelial barrier regulation, and cross-talk with the bacterial microbiota, fungi contribute to shaping the immunological tone of the host and may influence the course of the disease. Consequently, the gut mycobiome is emerging not only as a potential biomarker of disease progression^53^ but also as a modifiable component of the tumor–host interface that may be harnessed therapeutically.^54^ Furthermore, fungal dysbiosis has been associated with increased intestinal permeability, systemic inflammation, and metabolic disturbances, all of which can compromise the patient’s resilience to treatment and alter drug pharmacokinetics.^55^ These findings underscore the potential of targeting the gut mycobiome as a complementary strategy to enhance the precision and efficacy of cancer therapies. Additionally, there is growing evidence of fungal translocation, particularly of *Malassezia*, from the gut to distant organs, including the pancreas and liver.^56-58^ This process appears to facilitate tumor-promoting immune signals and highlights the functional significance of gut fungi in systemic cancer biology.^37^^,^^56^^,^^59^

In summary, gut fungal dysbiosis in cancer is characterized by decreased diversity, overrepresentation of pathobionts, ecological imbalance, and disrupted microbial cooperation, all converging on immune modulation and cancer progression. These insights provide a foundation for developing diagnostic and therapeutic strategies targeting the mycobiome.

## Gut mycobiome as a modulator and predictor of cancer immunotherapy response

In a recent study, Hu et al.^60^ demonstrated that specific characteristics of the gut mycobiome may serve as predictive biomarkers for the efficacy of ICIs, particularly anti-PD-1/PD-L1 therapies, highlighting a previously underappreciated role of fungi in shaping CRC treatment outcomes. By analyzing metagenomic data from nearly 600 cancer patients before treatment, the authors identified two distinct gut mycobiome-based enterotypes (distinct clusters or groups of microbial communities present in the human gut microbiome) strongly associated with clinical responses to immunotherapy. Favorable-type enterotype has been characterized by higher fungal and bacterial alpha diversity, a higher *Basidiomycota*/*Ascomycota* ratio, an increased presence of butyrate-producing bacteria, and metabolic pathways related to butyric acid and sugar/starch metabolism. Moreover, when externally validated on an additional cohort comprising 125 pan-cancer patients that received PD1 antibody treatment, identified fungal enterotypes were significantly associated with immunotherapy efficacy outcomes. Importantly, fecal microbiota transplantation (FMT) from favorable-type donors enhanced response to anti-PD-1/PD-L1 therapy. These findings are consistent with earlier research demonstrating that fungal overgrowth, particularly of *C. albicans,* can impair host immune modulation and diminish the success of FMT.^61^ Such evidence underscores how fungal dysbiosis may compromise microbiota-driven immune regulation and, by extension, influence the clinical effectiveness of cancer immunotherapies. Additionally, this microbiome configuration was correlated with enhanced cytotoxic CD8^+^ T cell infiltration in the tumor microenvironment (TME) and improved treatment outcomes.^60^ Multi-omics and single-cell RNA sequencing further confirmed stronger immune activation signatures in patients with the favorable fungal-bacterial profile.

The study of Huang et al. is another large-scale, multi-cohort investigation into the predictive power of the gut mycobiota in the context of immunotherapy.^62^ Using metagenomic data from 862 cancer patients across nine cohorts (treated with anti-PD-1, PD-L1, or CTLA-4 therapies), the authors identified distinct fungal signatures that distinguish responders from non-responders. Notably, fungal markers alone achieved higher predictive accuracy (AUC = 0.87) than bacterial markers (AUC = 0.83), and the combination of both kingdoms in a multi-kingdom model improved prediction further (AUC = 0.89). The fungal model also correlated with prolonged overall and progression-free survival in multiple cohorts. Specific fungal taxa, such as *Schizosaccharomyces octosporus*, were enriched in responders and were associated with beneficial metabolic functions, such as SCFA production, which is linked to enhanced anti-tumor immunity in some research.^63^^,^^64^ Additionally, responders showed distinct fungal–bacterial interaction networks.^62^ These interactions were largely absent or less coordinated in non-responders, suggesting that fungal–bacterial synergy may be important for shaping an immune environment conducive to ICI efficacy. Mechanistically, predicted responders exhibited a TME with increased expression of exhaustion markers (PD-1, PD-1L, CTLA-4, and TIM-3), indicating a pre-activated immune state more amenable to ICI response. Patients predicted to respond based on fungal markers are more likely to benefit from PD-1 and CTLA-4 blockades, as indicated by an enriched exhausted T cell signature marked by elevated expression of PD-1 and CTLA-4.^62^ These findings support the hypothesis that fungal dysbiosis in the gut may influence the efficacy of ICB and melanoma progression, highlighting the gut mycobiota as a potential modulator of immunotherapy outcomes and a source of therapeutic targets or prognostic biomarkers.

In line with these large-scale studies, more focused cohort analysis by Dora et al. revealed the prognostic relevance of specific gut fungal taxa in lung cancer patients undergoing ICI therapy.^65^ By integrating shotgun metagenomics and ITS sequencing with CT-based radiomics in advanced NSCLC, the authors demonstrated that intestinal overrepresentation of *Cortinarius davemallochii*, as well as fungi from the orders *Helotiales* and *Chaetosphaeriales,* and the class *Tremellomycetes* correlated with short overall survival (≤6 months). In contrast, *Hymenoscyphus immutabilis* and *Clavulinopsis fusiformis* were enriched in patients with high PD-L1–expressing tumors, indicating a potential link between specific fungal taxa and tumor immune checkpoint status. Members of the *Thelephoraceae* family were linked to immune-related toxicities, whereas *Cutaneotrichosporon cutaneum* and members of *Rozellomycota* were more prevalent in patients without iRAEs. Interestingly, more fungal than bacterial taxa were associated with poor outcomes, suggesting that fungal over-colonization may represent a negative modulator of ICI efficacy. These findings underscore the emerging importance of the gut mycobiome not only as a biomarker of immunotherapy response but also as a potential determinant of treatment-related toxicity.

Importantly, FMT experiments in murine models demonstrated that transfer from the donor with the favorable enterotype led to increased butyrate production, upregulation of anti-tumor immune pathways, and potentiation of anti-PD-1 efficacy.^60^ Work by Lam et al. emphasized that FMT efficacy is governed not only by bacterial composition but also by the fungal and viral constituents of the gut microbiota, which interact in complex and dynamic ways to modulate donor engraftment and therapeutic outcomes.^66^ Further supporting this multi-kingdom perspective, Zuo et al. reported that the gut virome and mucosal mycobiota undergo coordinated shifts in inflammatory diseases – alterations that may influence immune checkpoint pathways.^67^ These findings suggest that gut fungal states could indirectly modulate systemic immunity by priming mucosal and lymphoid tissues toward either pro-inflammatory or tolerogenic phenotypes. Key fungi, such as *Malassezia pachydermatis, Kalmanozyma brasiliensis, Pseudogymnoascus* sp*. VKMF-4246,* and *Thermothielavioides terrestris*, showed increased presence in recipients after FMT from favorable-type donors. Of note, fungal species found to be enriched in patients with favorable responses to immunotherapy, such as *Penicillium antarcticum*, *Aspergillus terreus*, and *T. terrestris*, are known to produce a range of glycoside hydrolase enzymes. These include *β*-galactosidases, *α*-L-arabinofuranosidases, and enzymes involved in the breakdown of plant-derived lignocellulose.^68-70^ Among them, *β*-galactosidases are particularly notable for their role in synthesizing lactose-derived compounds like galacto-oligosaccharides (GOSs).^71^ GOSs are recognized for their prebiotic effects, as they support the proliferation of beneficial gut bacteria, notably butyrate-producing species such as *Faecalibacterium prausnitzii,*^72-74^ which are themselves associated with improved responses to ICIs.^75-77^ Altogether, these findings suggest that the gut mycobiome serves not only as a biomarker of response but also as a functional modulator of cancer immunotherapy, likely through metabolic and immunological cross-talk with the bacterial microbiota.

## Host immune modulation by fungi

**Figure 1.**
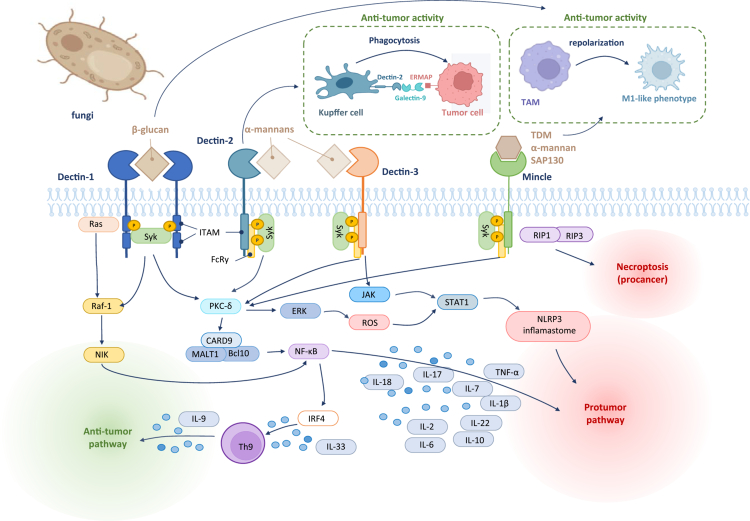
Schematic representation of the immunomodulatory pathways triggered by fungal components in the tumor microenvironment. This diagram illustrates the dual role of fungal ligands in modulating anti- and pro-tumor immune responses via C-type lectin receptors (CLRs) such as Dectin-1, Dectin-2, Dectin-3, and Mincle. Recognition of fungal components (e.g., *β*-glucans, *α*-mannans, TDM, SAP130) by CLRs on immune cells activates downstream signaling cascades involving Syk, CARD9, PKC-*δ*, and the NLRP3 inflammasome, leading to the production of cytokines such as IL-1β, IL-6, IL-9, IL-10, IL-17, and TNF-*α*. These pathways may promote either tumor-supporting inflammation (protumor pathway) or anti-tumor immunity through mechanisms such as phagocytosis, Th9 differentiation, or M1-like macrophage polarization.

Fungi modulate the host immune system predominantly via activation of innate immune pathways. Fungal PAMPs, including *β*-glucans, mannans, and chitin localized on the fungal wall, engage pattern recognition receptors (PRRs) such as Dectin-1, Dectin-2, Dectin-3, TLR2, NOD-like receptors (NLRs), retinoic acid-inducible gene I (RIG-I)-like receptors (RLRs), and the mannose receptor (MR).^78-81^ Distinct PRRs recognize specific PAMPs from different fungal species, e.g., Dectin-1 primarily binds *β*-glucans,^82^ chitin is bound by LysM domain 3,^83^ whereas mannans are recognized by Dectin-2, Dectin-3, Mincle, MR, and DCs-specific intercellular adhesion molecule 3 (ICAM3)-grabbing non-integrin (DC-SIGN).^81^^,^^84-86^ Binding PAMPs by PRRs triggers intracellular signaling cascades that lead to cytokine secretion and recruitment of immune effector cells. CLRs such as Dectin-1, Dectin-2, and Mincle activate the spleen tyrosine kinase - caspase recruitment domain-containing protein 9 (SYK-CARD9) signaling axis, which plays a central role in antifungal innate immunity by driving proinflammatory responses and promoting protective Th17 cell differentiation.^41^^,^^87-90^ Upon fungal recognition, SYK–CARD9 signaling induces NF-κB, extracellular signal-regulated kinase (ERK), p38 MAPK, and JNK activation, leading to the production of cytokines including IL-1β, IL-2, IL-6, IL-10, IL-17, IL-18, and tumor necrosis factor alpha (TNF-*α*), which collectively orchestrate downstream adaptive immune responses.^88^^,^^91-94^ Beyond this canonical SYK-dependent route, Dectin-1 can also signal through a SYK-independent pathway involving Raf-1 and NF-κB-inducing kinase (NIK), further modulating NF-κB activity.^95^ This parallel pathway is thought to integrate with SYK-mediated signaling to fine-tune DCs’ responses and facilitate Th cell differentiation. Through these combined mechanisms, fungal engagement of innate immunity can shape T cell priming and polarization, potentially modulating the effectiveness of immune checkpoint blockade (ICB) therapies. An overview of the immunomodulatory effects of fungi in cancer is presented in Table 1 and illustrated in Figure 1.

**Table 1.** Immunomodulatory effects of fungi in cancer.

PRRs	Immune cells	Effect on cancer	Immunomodulatory effect	Cancer types	Model	References
**Dectin** **-1**	macrophages	Pro-tumoral	differentiation of macrophages toward a tolerogenic phenotype; T cell dysfunction	PDA, gastric cancer	mouse PDA + human PDA samples	[96,97]
**Dectin** **-1**	MDSCs	Pro-tumoral	promotion of PGE₂ production; suppression of IL-22 binding protein expression	CRC	mouse AOM/DSS CRC + human CRC samples	[98]
**Dectin** **-1**	MDSCs	Pro-tumoral	MDSCs activation, reduction of T cell cytotoxicity, promotion of the accumulation of PD-1⁺ CD8⁺ T cells	lung cancer	mouse lung cancer + human samples	[99]
**Dectin** **-1**	MDSCs	Pro-tumoral	increased infiltration of Tregs and MDSCs, enhanced IL-1β production	OSCC	mouse OSCC + human samples	[100]
**Dectin** **-1**	macrophages	Pro-tumoral	M2 macrophage polarization	gliomas	human	[101]
**Dectin** **-1**	macrophages	Anti-tumoral	M-CSF–mediated downregulation of TLR4 and CD14	liver cancer	mouse + human	[102]
**Dectin** **-1**	macrophages	Anti-tumoral	reprogramming macrophages, enhancement of T cell responses	PDA	mouse PDA	[103]
**Dectin** **-1**	macrophages	Anti-tumoral	conversion of TAMs into M1-like phenotype via SYK–CARD9–Erk pathway	lung cancer, breast cancer, melanoma	mouse	[104]
**Dectin** **-1**	macrophages/NK cells	Anti-tumoral	interaction with MS4A4A in lipid rafts enhances NK activation and anti-metastatic responses	melanoma, lung cancer, renal cancer	mouse + human	[105]
**Dectin** **-1**	DCs	Anti-tumoral	upregulation of IL-33 via SYK/Raf-1/NF-κB, leading to Th9 differentiation and IL-9 production	unspecified	mouse, B16 and B16-OVA, MPC-11 cell lines,	[106–109]
**Dectin** **-1**	DCs	Anti-tumoral	plasma B cell differentiation, upregulation of costimulatory molecules, increased antibody production; increased CD19^+^ B cell infiltration in the TME, expansion of germinal center B cells	lung cancer	mouse + tumor cell lines	[110]
**Dectin** **-2**	Kupffer cells	Anti-tumoral	interaction with ERMAP and galectin-9 enhances phagocytosis of metastatic cells	liver cancer	mouse + human samples	[121]
**Dectin** **-2**	macrophages	Anti-tumoral	activation by BDC-3042 reprograms TAMs to a pro-inflammatory phenotype, enhancement of T cell infiltration	NSCLC, solid tumors		[122,123]
**Dectin** **-3**	macrophages	Anti-tumoral	promotes fungal clearance and epithelial repair during colitis; Dectin-3 deficiency exacerbates inflammation and tumorigenesis	CRC (colitis-associated)	mouse + fungi	[124]
**Dectin** **-3**	MDSCs	Pro-tumoral	activation of NLRP3 inflammasome via JAK–STAT1 and mitochondrial ROS pathways in response to *C. tropicalis*; promotes MDSC accumulation and suppresses T cell function	CRC	mouse + human + fungi	[33,34]
**Dectin** **-3**	macrophages	Anti-tumoral	inhibits glycolysis in TAMs, enhances CD4⁺ and CD8⁺ T cell infiltration; limits tumor growth	HCC	mouse + human	[125]
**Dectin** **-3**	macrophages	Pro-tumoral	deficiency promotes *C. albicans* overgrowth, increased IL-7 → IL-22 in ILC3s via AhR/STAT3 → tumor-promoting inflammation	CRC	mouse + human	[126]
**Mincle**	**macrophages**	Pro-tumoral	expressed on M2 TAMs; promotes immunosuppressive phenotype via SYK–NF-κB signaling; associated with reduced T cell infiltration and poor prognosis	lung cancer	mouse + human + LLC, B16F10 cell lines	[132]
**Mincle**	macrophages	Pro-tumoral	senses SAP130 from necrotic cells; activates RIP1/RIP3 signaling; suppresses recruitment of immunogenic myeloid cells; facilitates immune evasion	PDA	mouse + human PDA cell lines	[133]
**Mincle**	**macrophages**	**Anti-tumoral**	activation with synthetic ligand TDB induces GM-CSF–dependent TAM repolarization toward M1-like phenotype; enhances anti-tumor immunity	Preclinical model	mouse	[134]

### Dectin-1 – a key fungal-sensing receptor in cancer immunity

Dectin-1 (CLEC7A), a C-type lectin receptor, exhibits dual roles in cancer immunity, acting as either an immunosuppressive or immunostimulatory agent depending on the tumor context and ligand availability. Recent studies have elucidated its capacity to either enhance anti-tumor immune responses or diminish them, in both cases through various mechanisms. Given its impact on the immune system, including T cell function, myeloid cell behavior, and cytokine signaling, Dectin-1 represents a promising therapeutic target, either alone or in combination with ICIs.

#### Dectin-1 as an immunosuppressive agent

Multiple studies have highlighted the immunosuppressive role of Dectin-1 signaling in the TME, particularly in PDA and other malignancies. Daley et al. demonstrated that Dectin-1 is highly expressed on tumor-associated macrophages (TAMs) in PDA, where it acts as an immunosuppressive oncogene. This signaling promotes the differentiation of macrophages toward a tolerogenic phenotype and impairs both CD4⁺ and CD8⁺ T cell function, facilitating immune evasion.^96^ This pathway is activated by Galectin-9, a non-fungal ligand, suggesting that Dectin-1's immunomodulatory effects extend beyond fungal recognition. Blocking Galectin-9 led to marked tumor regression and synergized with anti-PD-1 therapy, suggesting that targeting Dectin-1 or its ligand may enhance immunotherapy efficacy in PDA.^96^ Similarly, in gastric cancer, Dectin-1-positive TAMs are associated with T cell dysfunction and poor patient survival.^97^ Blocking Dectin-1 in this context reprograms TAMs and enhances the efficacy of anti-PD-1 therapy.^97^

Beyond macrophages, Dectin-1 is also expressed on myeloid-derived suppressor cells (MDSCs), contributing to immunosuppression in the TME. In CRC, Dectin-1 signaling in MDSCs promotes prostaglandin E₂ (PGE₂) production, suppressing IL-22 binding protein expression, facilitating tumorigenesis. Blocking Dectin-1 reduces MDSC-mediated immunosuppression and tumor progression.^98^ In lung cancer, Dectin-1-mediated recognition of *Aspergillus sydowii* via *β*-glucan/Dectin-1/CARD9 signaling induces MDSCs activation, reduces T cell cytotoxicity, and promotes the accumulation of PD-1⁺ CD8⁺ T cells, further contributing to immune suppression.^99^ Similarly, in oral squamous cell carcinoma (OSCC), Dectin-1 expression was associated with increased infiltration of regulatory T cells (Tregs) and MDSCs and enhanced IL-1β production – an effect exacerbated with age.^100^ Genetic ablation of Dectin-1 in mice reversed these effects, reducing intratumoral immunosuppressive cell infiltration and cytokine expression. Gliomas studies further link high Dectin-1 expression to M2 macrophage polarization and poorer prognosis, suggesting that Dectin-1 inhibition could enhance anti-tumor immunity.^101^

Taken together, these findings highlight a unifying mechanism across multiple tumor types, where Dectin-1 signaling fosters an immunosuppressive TME by driving tolerogenic macrophage/MDSC polarization, promoting PGE₂ and IL-1β production, and increasing checkpoint expression, all of which converge on dampening T cells cytotoxicity.

#### Dectin-1 as an immunostimulatory agent

In contrast, Dectin-1 signaling can also promote anti-tumor immunity when activated in distinct cellular or therapeutic contexts. Dectin-1 expression is upregulated in hepatic fibrosis and liver cancer, and its deletion exacerbates disease progression. Mechanistically, Dectin-1 suppresses TLR4 signaling in hepatic inflammatory and stellate cells, partly via M-CSF–mediated downregulation of TLR4 and CD14. Dectin-1–deficient mice show heightened cytokine responses and reduced survival in LPS-induced sepsis, while Dectin-1 activation is protective.^102^ Moreover, combining Dectin-1 activation via systemic *β*-glucan therapy with CD40 agonists has shown synergistic effects in overcoming resistance to checkpoint inhibitors in PDA models. This coactivation reprograms macrophages and enhances T cell responses, leading to tumor regression and immunological memory.^103^

Additionally, *β*-glucan, a natural Dectin-1 ligand, can convert immunosuppressive TAMs into an M1-like phenotype, promoting anti-tumor immunity. This effect is mediated through the Dectin-1-SYK-CARD9–Erk signaling pathway.^104^ Research by Mattiola et al. demonstrated that Dectin-1 interacts with the tetraspan molecule MS4A4A within macrophage lipid rafts. This interaction is crucial for activating natural killer (NK) cells and the subsequent resistance to cancer metastasis. Without MS4A4A, Dectin-1 signaling is impaired, leading to reduced production of proinflammatory mediators and diminished NK cell-mediated anti-tumor activity.^105^ Dectin-1 activation in DCs has been shown to upregulate the expression of IL-33 via the SYK/Raf-1/NF-κB signaling pathway, which in turn enhances the expression of interferon regulatory factor 4 (IRF4).^106^ This cascade leads to increased IL-33 production, a cytokine known to play a significant role in anti-tumor immunity.^106^ Furthermore, IL-33 contributes to the induction of Th9 cells, a subset of Th cells that produce IL-9 and have potent anti-tumor effects.^107^^,^^108^ Blocking IL-33 or its receptor ST2 inhibits Th9 differentiation from CD4^+^ T cells, underscoring the importance of this pathway in anti-tumor responses.^108^ Dectin-1-activated DCs have been identified as powerful inducers of Th9 cells. Upon activation by *β*-glucan ligands, Dectin-1 stimulates DCs to upregulate costimulatory molecules such as OX40L and TNFSF15, essential for Th9 differentiation. These Th9 cells produce IL-9, contributing to robust anti-tumor immunity. *In vivo* studies have shown that immunization with Dectin-1-activated DCs leads to significant tumor regression, primarily mediated by Th9 cells and IL-9 production.^107^^,^^109^ A 2025 study by Bai et al. revealed that *β*-glucan-induced Dectin-1 activation promotes the differentiation of plasma B cells, co-stimulatory molecule expression, and Ig production, enhancing anti-tumor immune responses.^110^ In mouse models of lung cancer, *β*-glucan combined with anti-PD-1 therapy increased CD19^+^ B cell infiltration in the TME, expanded germinal center B cells, and improved anti-tumor efficacy. This finding extends the known immunostimulatory roles of Dectin-1 beyond T cell-mediated immunity, highlighting its potential in orchestrating comprehensive anti-tumor responses involving multiple immune cell types.^110^

In summary, Dectin-1 can amplify anti-tumor immunity through coordinated reprogramming of multiple immune compartments such as macrophages, DCs, NK cells, Th9 cells, and B cells, largely via SYK–CARD9–NF-κB–dependent pathways and ligand-driven activation by *β*-glucans.

#### Context-dependent role of Dectin-1 in cancer

**Figure 2.**
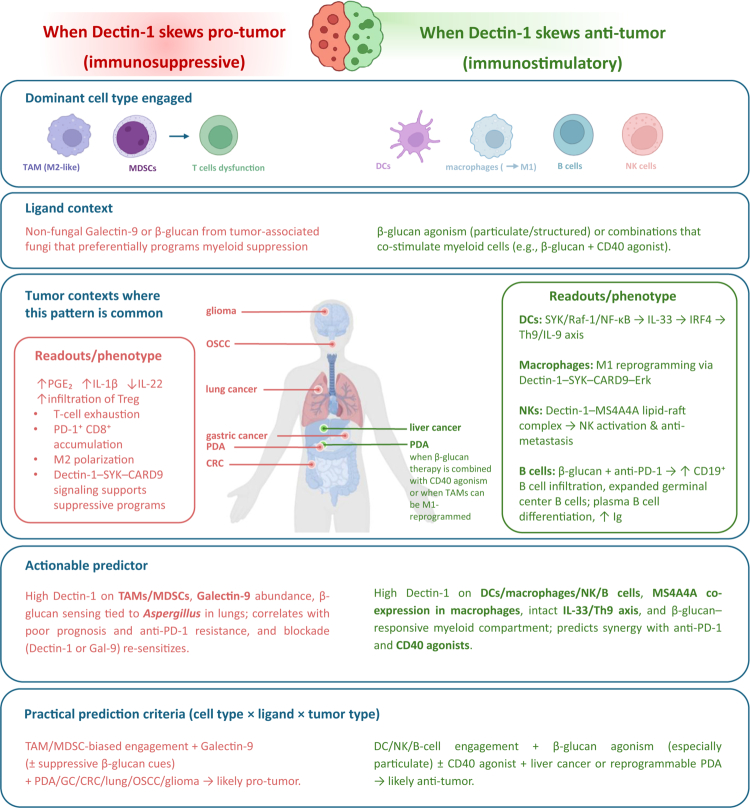
Context-dependent roles of Dectin-1 in cancer. Schematic overview illustrating how Dectin-1 activation can skew either toward a pro-tumor (left, immunosuppressive) or anti-tumor (right, immunostimulatory) response depending on ligand type, dominant immune cell engagement, and tumor context. On the pro-tumor side, Dectin-1 signaling in TAMs/MDSCs promotes PGE₂, IL-1β, Treg infiltration, reduced IL-22, CD8⁺ T cell exhaustion, and M2 polarization, commonly observed in PDA, gastric cancer, CRC, lung cancer, OSCC, and glioma. On the anti-tumor side, particulate *β*-glucan or *β*-glucan + CD40 agonist promote activation of DCs, macrophages, NK cells, and B cells, leading to IL-33/Th9/IL-9 signaling, M1 reprogramming, NK cytotoxicity, and B cell infiltration with germinal center expansion and antibody production, observed in liver cancer and reprogrammable PDA. Actionable predictors include expression levels of Dectin-1, Galectin-9, MS4A4A, and IL-33/Th9 activity, which may guide therapeutic interventions and identify synergy with anti-PD-1 therapy.

The impact of Dectin-1 on tumor immunity is highly context-dependent. The same receptor can induce either tolerogenic pathways (via TAM/MDSC polarization and checkpoint induction) or immunostimulatory cascades (via DC/Th9 priming, NK and B cell activation), with the net outcome dictated by ligand type and cellular context. When engaged predominantly on TAMs and MDSCs, and triggered by ligands such as Galectin-9 or tumor-associated *β*-glucans, Dectin-1 signaling drives immunosuppressive reprogramming, leading to PGE₂ and IL-1β production, T cell dysfunction, and resistance to checkpoint blockade. Conversely, when activated on DCs, NK cells, or B cells by structured *β*-glucans, Dectin-1 initiates SYK–CARD9–NF-κB signaling cascades that promote M1 macrophage polarization, IL-33/Th9 and IL-9 axis activation, NK cell–mediated cytotoxicity, and B cell differentiation, thereby amplifying anti-tumor immunity. Thus, who senses (TAM/MDSC vs DC/NK/B), what is sensed (Gal-9 vs *β*-glucan and its form), and where (tumor type: PDA/GC/CRC/lung/glioma vs liver or *β*-glucan-primable settings) together seem to forecast whether Dectin-1 tilts toward tumor promotion or tumor control (Figure 2).

Box. Context matters for Dectin-1.

**Table:** 

**Pro-tumor (immunosuppressive):** • **Cell type: TAMs, MDSCs** • **Ligand:** Galectin-9, suppressive *β*-glucans • **Tumor:** PDA/GC/CRC/lung/glioma • **Effect:** T cell dysfunction, PGE₂/IL-1β, resistance to anti-PD-1	**Anti-tumor (immunostimulatory):** • **Cell type:** DCs, NK cells, B cells • **Ligand:** Structured *β*-glucans ( ± CD40 agonism) • **Tumor:** liver/PDA • **Effect:** M1 polarization, IL-33/Th9/IL-9 axis, NK cytotoxicity, B cell activation, improved ICI efficacy
**Who** senses **what**, in which tumor context (**where**), **dictates whether Dectin-1 suppresses or amplifies anti-tumor immunity**.

#### Clinical implications of targeting Dectin-1 in cancer

In acute myeloid leukemia (AML), Dectin-1 has emerged as a potential biomarker for therapeutic responsiveness. A 2024 study found that AML cells exhibiting high expression of Dectin-1 and CD14, indicative of monocytic differentiation, were more sensitive to the MEK inhibitor trametinib.^111^ Although authors did not explain the role of Dectin-1 in details, mechanistically, Dectin-1 engagement in monocyte-derived cells can modulate MAPK/ERK signaling through SYK–CARD9 pathways, potentially influencing responsiveness to MEK inhibition. This suggests that Dectin-1 expression could serve as a predictive marker for trametinib sensitivity in AML patients. Furthermore, genetic variations in Dectin-1 have been associated with increased susceptibility to invasive fungal diseases in AML patients undergoing induction chemotherapy.^112^ Specifically, the rs7309123 G/G genotype was linked to a higher risk of developing pulmonary infections, including invasive fungal diseases. Although this SNP is located in an intron and as such does not alter the protein sequence directly, it may affect Dectin-1 expression or splicing, potentially modulating antifungal immune responses and increasing vulnerability to infections during immunosuppressive treatment. These findings underscore the importance of Dectin-1 in host defense mechanisms and its potential as a target for therapeutic intervention.

In melanoma models, stimulating Dectin-1 with curdlan significantly reduced tumor progression.^113^ Dectin-1 expression was associated with an activated myeloid cell phenotype in the TME, characterized by expression of co-stimulatory molecules such as CD80 and CD86 and antigen presentation molecules (MHC-I and MHC-II). Overexpression of these molecules is likely to enhance anti-tumor immune responses. Mechanistically, Dectin-1 activation in myeloid cells leaded to increased production of pro-inflammatory cytokines such as IL-12 and TNF-*α*, which in turn promoted the recruitment and activation of effector T cells within the TME, thereby amplifying anti-tumor immunity. Consistently, RNAseq analyses of bone marrow–derived neutrophils indicated that curdlan treatment induced a shift toward an activated phenotype, characterized by upregulation of pro-inflammatory cytokines (IL-23a, IL-1β, IL-6, IL-12a, IL-1R2), chemokines (CXCL10, CCL3, CCL4), and co-stimulatory molecules (CD80, CD86, CD14), along with downregulation of suppressive markers (TGF-β2, CCL22, CD209, MRC1/CD206, CD163). These changes suggest that Dectin-1 activation enhances immune activation, pro-inflammatory signaling, and metabolic regulation, contributing to a protective anti-tumor mechanism in melanoma models. Notably, this Dectin-1–mediated mechanism appeared to be tumor type–specific, as similar effects were not observed in CRC models.

In a study by Wattenberg et al,^103^ systemic administration of *β*-glucan was combined with agonistic anti-CD40 antibody therapy in mouse models of PDA, a tumor type notably resistant to ICIs. This combinatorial treatment led to complete tumor eradication and the development of immunological memory. Mechanistically, Dectin-1 activation by *β*-glucan, together with CD40 signaling, reprogrammed intratumoral macrophages via interferon gamma (IFN-*γ*)–dependent pathways, promoting a tumoricidal microenvironment. Remarkably, this anti-tumor effect was independent of classical T cell–mediated cytotoxicity and did not rely on PD-1/PD-L1 or CTLA-4 blockade, underscoring an alternative route to effective immunosurveillance. The therapeutic synergy between fungal *β*-glucans and CD40 agonism demonstrates that components of the mycobiota, or their molecular analogs, can be leveraged to overcome tumor-induced immunosuppression, particularly in settings where conventional checkpoint inhibition fails. These results suggest that fungal ligands such as *β*-glucans may serve not only as immune adjuvants but also as central modulators of myeloid-driven anti-tumor responses. Collectively, these studies illuminate the critical role of Dectin-1 in immune regulation and its potential as a biomarker and therapeutic target in cancer.

### Dectin-2 CLR family

Dectin-2, along with Dectin-3 and Mincle, belongs to the Dectin-2 family of CLRs. These receptors possess short cytoplasmic tails and a single extracellular carbohydrate recognition domain (CRD). They associate with the immunoreceptor tyrosine-based activation motif (ITAM)-containing Fc receptor *γ*-chain (FcRγ) to initiate intracellular signaling cascades.^114-116^ Upon ligand binding, these receptors activate SYK, leading to the formation of the CARD9-BCL10-MALT1 (CBM) complex. This complex subsequently activates the NF-κB pathway, resulting in the production of pro-inflammatory cytokines and chemokines.^117^^,^^118^

#### Dectin-2 – from *α*-mannan recognition to clinical applications in cancer

Dectin-2 primarily recognizes *α*-mannans in a calcium-dependent manner and can form heterodimers with Dectin-3.^84^^,^^85^ These heterodimers exhibit enhanced binding to *α*-mannans, triggering more potent inflammatory responses compared to their respective homodimers.^85^ Through activation of the SYK–CARD9 signaling cascade, Dectin-2 induces IL-17 production in response to *C. albicans*, bridging innate and adaptive antifungal immunity^119^). Beyond classical fungal defense, Dectin-2 plays an emerging role in cancer immunity. In the liver, Dectin-2 plays a crucial role in mediating the phagocytic activity of Kupffer cells, the resident macrophages of the liver.^120^ Kimura et al. demonstrated that Dectin-2 expression on Kupffer cells enhances their ability to phagocytose cancer cells, thereby suppressing liver metastasis. Further mechanistic insights revealed that this phagocytic capacity depends on the formation of a bridging complex involving erythroid membrane-associated protein (ERMAP) expressed on tumor cells and galectin-9 on Kupffer cells.^121^ This complex delivers an “eat me” signal to Kupffer cells, promoting the clearance of metastatic cancer cells. Notably, patients with tumors exhibiting low ERMAP expression showed a higher incidence of liver metastasis, underscoring the clinical relevance of this pathway.^121^ While these findings highlight the tumor-suppressive role of Dectin-2 in the liver, it remains unclear whether tumor cells can exploit this mechanism to evade immune surveillance. Moreover, the upstream activators that trigger the Dectin-2 complex in the liver microenvironment and the potential involvement of intra-tumoral fungi in this process warrant further investigation.

Despite these uncertainties, and considering the evidence for Dectin-2-mediated tumor control, Bolt Biotherapeutics has developed BDC-3042, a first-in-class agonist antibody targeting Dectin-2.^122^ Preclinical studies have shown that BDC-3042 can reprogram TAMs from an immunosuppressive to a pro-inflammatory phenotype, enhancing antigen presentation and promoting T-cell-mediated anti-tumor responses. Currently, BDC-3042 is undergoing a Phase 1/2 clinical trial to evaluate its safety and efficacy in patients with metastatic or unresectable solid tumors, including NSCLS. However, results from early-phase clinical trials showed that therapy was well-tolerated and exhibited immunostimulatory effects, including modulation of immune cell frequencies and activation markers, supporting further investigation into C-type lectin receptor-targeted therapies.^123^

#### Dectin-3 as a context-dependent regulator of fungal sensing and tumor immunity

Dectin-3, another C-type lectin receptor, is less studied compared to Dectin-1 and Dectin-2, but emerging research suggests its involvement in cancer immunity. Dectin-3 functions as a PRR in myeloid cells, recognizing *C. albicans* and *Cryptococcus* species.^119^ While specific clinical trials targeting Dectin-3 are limited, studies indicate its role in modulating immune responses within the TME. In CRC, Dectin-3 has been implicated in modulating immune responses via its interaction with fungal pathogens.^124^ Mice lacking Dectin-3 are more susceptible to DSS-induced colitis, associated with overgrowth of *C. tropicalis.*^124^ This phenotype results from impaired macrophage-mediated fungal clearance and defective cytokine responses, which in turn hinder epithelial repair. Antifungal treatment ameliorated colitis in Dectin-3–deficient mice, underscoring the importance of fungal sensing in gut immune regulation.

Dectin-3 also influences the function of MDSCs, which are known to suppress anti-term immunity. Studies have demonstrated that in CRC, overgrowth of *C. tropicalis* promotes the accumulation of MDSCs, fostering an immunosuppressive TME and accelerating tumor progression.^34^ Experimental models have shown that fungal overgrowth exacerbates CRC in CARD9-deficient mice, whereas antifungal treatment with fluconazole reduces MDSC infiltration and suppresses tumor development.^34^ Research indicates that *C. tropicalis* promotes CRC progression by activating NLRP3 inflammasome signaling in MDSCs via Dectin-3.^33^ This activation depends on enhanced glycolysis driven by glycogen metabolism and requires both priming and activation signals: transcriptional upregulation of inflammasome components via JAK–STAT1 signaling, and mitochondrial reactive oxygen species (ROS) as the second signal. The inhibition of NLRP3 signaling suppressed tumor growth and MDSC infiltration in an AOM/DSS-induced CRC model. Elevated STAT1 and NLRP3 expression in MDSCs from human CRC samples supports the clinical relevance of the STAT1–NLRP3 axis as a potential therapeutic target.

In the context of HCC, Dectin-3 appears to exert a tumor-suppressive effect. A study utilizing Dectin-3 knockout mice revealed that the absence of this receptor led to enhanced tumor growth, accompanied by increased macrophage glycolysis and reduced infiltration of CD4⁺ and CD8⁺ T cells.^125^ Mechanistically, Dectin-3 appears to inhibit tumor progression by regulating glycolytic metabolism in TAMs, thereby modulating the immune environment within the tumor. *In vitro* studies showed that Dectin-3-deficient macrophages promoted proliferation and inhibited apoptosis of H22 hepatoma cells.^125^ Moreover, mice lacking Dectin-3 exhibit increased tumorigenesis in CRC and a higher burden of *C. albicans* upon chemical induction.^126^ This elevated fungal load triggers glycolysis in macrophages, leading to the secretion of IL-7. IL-7, in turn, induces IL-22 production in group 3 innate lymphoid cells (ILC3s) via the aryl hydrocarbon receptor and STAT3 pathways.^126^ This cascade promotes tumorigenesis, highlighting the critical role of Dectin-3 in regulating immune responses to fungal components in the gut. *C. albicans* is also the primary driver of human antifungal Th17 responses, with Th17 cells targeting other fungi arising through cross-reactivity.^38^ Intestinal inflammation promotes expansion of both *C. albicans*-specific and cross-reactive Th17 cells.

The dualistic nature of Dectin-3's role in cancer underscores the importance of context when considering therapeutic strategies. Targeting Dectin-3 or its downstream signaling pathways could offer novel approaches to modulate the immune response in cancer. However, given its varying effects depending on the tumor type and microenvironment, personalized approaches would be essential.

#### Mincle – a checkpoint of myeloid-driven immunosuppression and a target for macrophage repolarization

Mincle (macrophage-inducible C-type lectin, CLEC4E) is a PRR that recognizes various PAMPs, including *α*-mannan, SAP130, and trehalose-6,6′-dimycolate (TDM).^115^^,^^127^^,^^128^ Mincle regulates cytokine production in response to *Candida* species by activating the SYK–CARD9 signaling pathway and protein kinase Cδ (PKC-*δ*), playing a key role in the clearance of *C. tropicalis* during systemic infection.^129^^,^^130^ While its expression is relatively low in myeloid cells, Mincle has been implicated in modulating the TME and influencing cancer progression.^115^ Moreover, its expression in myeloid cells is inducible and regulated by other CLRs, particularly Dectin-3.^116^^,^^131^ In lung cancer, Mincle is predominantly expressed on M2-polarized TAMs, where it promotes an immunosuppressive phenotype via the SYK–NF-κB signaling axis.^132^ Elevated Mincle expression correlates with poor prognosis, reduced T cell infiltration, and enhanced tumor progression.^132^ Mincle also plays a critical role in PDA, where its interaction with SAP130 released during necroptosis activates RIP1/RIP3-dependent signaling, suppresses immunogenic myeloid cell recruitment, and facilitates tumor immune evasion.^133^ Deletion of Mincle in murine PDA models leads to decreased tumor growth and a more pro-inflammatory TME, characterized by increased CD8⁺ T cell infiltration. Notably, Mincle signaling can have context-dependent effects. For example, Trehalose dibehenate (TDB), a synthetic Mincle ligand, has been shown to promote TAM repolarization toward a pro-inflammatory M1-like phenotype through GM-CSF–dependent pathways, enhancing anti-tumor immunity in preclinical models.^134^ These findings indicate that Mincle not only senses fungal and necrotic cell ligands but also shapes the immune landscape in tumors. Its dual role – as a promoter of immunosuppression or a potential target for macrophage reprogramming – makes it a promising candidate for novel immunotherapeutic strategies.^135^^,^^136^

## Associations between immune checkpoint molecules and mycobiota

Increasing evidence suggests that the mycobiome plays a significant role in shaping immune responses to ICIs, although this area remains less explored compared to bacterial influences. The mycobiome may influence therapy outcomes both directly, through interactions with the host immune system, and indirectly by modulating bacterial community structure and function. The level of key immune checkpoint molecules, including PD-1, PD-L1, and CTLA-4, reflects the immunological landscape of the TME and is a central determinant of the efficacy of ICB therapies. Growing evidence suggests that tumor-associated fungi and antifungal immunity contribute to modulating these checkpoint pathways, particularly through CLRs. An overview of interactions between fungal components, CLR signaling, and immune checkpoint molecules is summarized in Table 2.

**Table 2.** Interactions between fungal components, CLR signaling, and immune checkpoint molecules.

Immune Checkpoint Molecule	Fungal Component/Organism/Ligand	CLR Involved	Immune Cell Type(s)	Mechanism of Action/Outcome	Implications for ICB Therapy	Model	References
**PD-L1**	*β*-glucan (from *C. albicans*)	Dectin-1	Neutrophils	nuclear translocation of PD-L1, secretion of CXCL1/2 → impaired migration on neutrophils, immunosuppression	PD-L1 blockade enhances neutrophils activity	mouse + human + fungi	[137]
**PD-L1, others**	Curdlan	Dectin-1	Neutrophils, CD4⁺ T cells	ROS-dependent suppression of T cells; SYK-CARD9–mediated PD-L1 induction (autoimmune setting)	-	mouse	[138]
**PD-L1**	Curdlan	Dectin-1	MDSCs	↓ PD-L1 in MDSCs (anti-tumor)	Modulation of Dectin-1 may enhance T cell responses while limiting suppressive MDSCs	human	[139]
**PD-L1**	*A. fumigatus*	CLR (unspecified)	DCs	activation of Wnt/β-catenin pathway → ↑ PD-L1, ↑ Tregs → immunosuppression	Potential resistance mechanism; targetable via CLR pathway inhibitors	human + fungi	[140]
**PD-L1**	*β*-glucan (WGP)	Dectin-1	Macrophages, DCs, MDSCs, T cells (indirect)	↑ infiltration of DCs and macrophages, ↓ frequency of MDSCs, ↓ proliferation of CD4^+^ T cells and Tregs in the tumor, ↓ PD-L1 in macrophages, ↑ PD-L1 in DCs,	Combination therapy (*β*-glucan + anti-PD-1/PD-L1) improves ICB responsiveness	mouse + tumor cell lines	[141]
**PD-L1**	*P. brasiliensis* yeasts	Dectin-1, TLR2, TLR4	M-MDSCs, PMN-MDSCs	Dectin-1 deficiency → ↓ PD-L1⁺ M-MDSCs accumulation *in vivo* → altered trafficking or survival of MDSCs + ↓ IL-10, ↓ nitrotyrosine + ↓ suppression of CD4^+^ and CD8^+^ T lymphocytes TLR2/TLR4 deficiency → ↑ PD-L1⁺ PMN-MDSCs + ↓ IL-10^+^ M-MDSCs + nitrotyrosine + ↓ CD4⁺ suppression	PRRs contribute to the suppressive activity of MDSCs by inducing the expression of immunosuppressive molecules; Dectin-1 influences immune cell composition rather than direct PD-L1 expression	mouse + fungi	[142]
**PD-L1**	*S. cerevisiae*, *Candida spp.*	Not CLR-dependent	Macrophages	PD-L1 binds fungal Rpl20b in phagosomes → IL-10 production → immune suppression	Highlights non-CLR mechanisms by which fungi modulate checkpoint activity	cell lines + primary cells	[143]
**CTLA-4**	—	Dectin-1	Tregs	Dectin-1 promotes Treg differentiation (αβ/γδ) even without TGF-β; deficiency worsens CTLA-4 haploinsufficiency phenotype	Dectin-1 acts as a modifier gene; CLR variants may influence ICB response or toxicity	mouse + human	[150]
**CTLA-4/Costim. molecules**	Curdlan	Dectin-1, TLR4	DCs	co-stimulation → ↑ MHC-I/II, CD40, CD80, CD86 → improved antigen presentation and T cell priming; ↑ IL-12, IL-1β, TNF-*α*, IFN-β	Mycobiota may enhance efficacy of CTLA-4 blockade via costimulatory molecule induction	mouse	[151]

### Fungal recognition rewires PD-L1–mediated suppression

Recent studies have highlighted the nuanced role of Dectin-1 signaling in regulating PD-L1 expression across various myeloid cell types, with implications for immune modulation in both infectious and tumor contexts. Activation of Dectin-1 by *β*-glucans, especially those derived from *C. albicans*, has been shown to upregulate PD-L1 in neutrophils by promoting nuclear translocation of PD-L1 and the subsequent secretion of CXCL1 and CXCL2.^137^ This mechanism results in impaired neutrophil migration and enhanced accumulation in the bone marrow, contributing to a localized immunosuppressive phenotype.^137^ Neutrophil-specific PD-L1 deficiency or its pharmacological blockade enhances neutrophil release into circulation, improving antifungal immunity. Thus, the Dectin-1/PD-L1 axis acts as a negative regulator of antifungal responses and represents a potential therapeutic target. A similar immunoregulatory pathway has been observed in autoimmune settings, such as experimental autoimmune encephalomyelitis, where Dectin-1 signaling suppresses CD4⁺ T cell responses via a ROS-dependent manner, followed by PD-L1 and other immune checkpoint induction via SYK-CARD9 signaling.^138^

Mashhouri et al. reported co-localization of Dectin-1 and PD-L1 in tumor-infiltrating myeloid cells in melanoma.^113^ Stimulation with curdlan (a Dectin-1 ligand) increased the production of pro-inflammatory cytokines and immune cell infiltration, leading to tumor growth suppression. Notably, curdlan-mediated Dectin-1 activation reduced PD-L1 levels in MDSCs, indicating a potential anti-tumor role of Dectin-1.^139^ On the other hand, Karnam et al. demonstrated that *A. fumigatus*, via CLR signaling, activates the Wnt/β-catenin pathway in DCs, leading to PD-L1 upregulation and enhanced Treg-mediated immunosuppression.^140^ These findings expand the functional axis of fungal–CLR–PD-L1 regulation beyond neutrophils and macrophages to broader immune cell networks within the TME. The differential regulation of PD-L1 by Dectin-1 in MDSCs and DCs underscores the importance of context in immune modulation. Understanding these nuances is crucial for developing targeted therapies that can modulate the TME effectively. For example, strategies that inhibit Dectin-1 signaling in MDSCs may reduce their immunosuppressive functions, while promoting Dectin-1 activation in DCs could enhance their ability to stimulate anti-tumor T cell responses.

In line with this, a 2024 preclinical and clinical study demonstrated that whole glucan particle (WGP) *β*-glucan enhances the efficacy of PD-1/PD-L1 ICB therapy.^141^ In mouse tumor models, co-administration of *β*-glucan and anti-PD-1/PD-L1 antibodies resulted in increased recruitment of immune effector cells, improved regulation of T cell activation versus tolerance, and delayed tumor progression. Importantly, this combination also prolonged progression-free survival in cancer patients who had previously developed resistance to PD-1/PD-L1 therapy, suggesting that *β*-glucan can reverse immune escape by reprogramming innate and adaptive responses. These findings position *β*-glucan as a promising immune adjuvant that modulates checkpoint responsiveness, potentially through its impact on Dectin-1–mediated PD-L1 regulation.

A 2024 study by Kaminski et al. further investigated the role of Dectin-1, TLR2, and TLR4 in PD-L1 expression in regulating the production of immunosuppressive molecules by MDSCs during pulmonary fungal infection.^142^ Although Dectin-1 deficiency did not significantly change PD-L1 levels on MDSCs *in vitro*, it led to a reduction of PD-L1⁺ monocytic MDSCs (M-MDSCs) *in vivo*, suggesting Dectin-1 may influence the survival or trafficking of PD-L1-expressing M-MDSCs rather than PD-L1 direct expression. Dectin-1, TLR2, and TLR4 also reduced the expression of IL-10 and nitrotyrosine.^142^ Additionally, Li et al.^143^ revealed a novel mechanism whereby PD-L1 contributes to innate sensing of fungal pathogens: PD-L1 is selectively enriched in macrophage phagosomes containing *S. cerevisiae* and *Candida* spp. and directly binds the fungal ribosomal protein Rpl20b, leading to the anti-inflammatory cytokine IL-10 production independently of canonical PRRs signaling. Both genetic depletion of Rpl20b in yeast and functional blockade of PD-L1 on host cells resulted in diminished IL-10 responses, confirming the specificity and functional relevance of this interaction. This adds another layer to the immunoregulatory role of PD-L1 in the context of fungal exposure and raises the possibility that fungal components of the mycobiota could impact responsiveness to ICIs.

Altogether, these findings support the notion that *β*-glucans act as immune modulators that enhance PD-1/PD-L1 blockade primarily through trained immunity (TI)-driven myeloid reprogramming and context-dependent PD-L1 ‘rewiring’ on myeloid cells.

*β*-Glucans engaging Dectin-1 (SYK–CARD9 axis) induce epigenetic and metabolic reprogramming of monocytes, macrophages, and DCs, shifting them toward a pro-inflammatory, antigen-presenting phenotype that improves T cell priming and effector function.^137^^,^^138^^,^^142^ Clinically, this manifests as increased effector infiltration, higher inflammatory cytokine competence upon secondary challenge, and improved responsiveness to ICIs.^141^ TI also helps explain durable benefits observed after short *β*-glucan exposure. As for the ‘rewiring’ mechanism, CLR engagement also modulates PD-L1 at the level of expression, localization (including nuclear pools), and cell-subset distribution.^137^^,^^139^^,^^142^^,^^143^ In tumors, *β*-glucans can reduce PD-L1 on suppressive MDSCs while enhancing pro-inflammatory outputs from DCs;^139^^,^^141^ in other contexts (e.g., neutrophils or DCs under certain stimuli), PD-L1 can be transiently upregulated.^137^^,^^138^^,^^143^ The net tumor-context effect is predicted to lower myeloid-derived suppression and heighten ICI sensitivity.

It is worth to mention that CR3/complement–mediated mechanisms may also contribute to enhanced checkpoint therapy, although likely as a secondary effect. *β*-glucans can allosterically activate CR3 (CD11b/CD18) and shape complement biology, which can influence antitumor immunity^144^^,^^145.^ However, given the limited Fc effector engagement of PD-1/PD-L1 antibodies compared to some other antagonistic monoclonal antibodies (mABs),^146^ these mechanisms are likely supportive rather than primary, potentially contributing through CR3-dependent cytotoxicity or iC3b-opsonization in specific contexts.

Future trials could evaluate these mechanisms using complementary approaches. For TI, *ex vivo* functional assays of patient monocytes, macrophages, and DCs, combined with epigenetic, transcriptional, and metabolic profiling, can reveal myeloid reprogramming. Context-dependent PD-L1 rewiring can be assessed by measuring PD-L1 expression and localization across myeloid subsets, functional co-cultures with T cells, and spatial mapping within tumors. Complement/CR3-mediated contributions can be monitored via CR3 activation and iC3b opsonization assays, together with serum complement profiling. Integrated, longitudinal biomarker analyses, combining single-cell multi-omics, functional readouts, and soluble mediators, will allow correlations between mechanistic changes and clinical outcomes such as immune response, tumor progression, and therapy durability.

### CTLA-4 and other ICIs

In addition to PD-1/PD-L1, CTLA-4, a critical checkpoint molecule expressed primarily on Tregs, has also been implicated in fungal immune interactions.^147^ CTLA-4 competes with CD28 for binding to CD80/CD86, dampening T cell activation and promoting immune tolerance.^148^^,^^149^ Recent studies have highlighted the multifaceted role of Dectin-1 in immune regulation, particularly in the context of CTLA-4 haploinsufficiency and AML. Turnbull et al. identified a patient with immune dysregulation, autoimmunity, and lymphoproliferation who carried a maternally inherited pathogenic CTLA4 variant and a paternally inherited rare loss-of-function missense variant in CLEC7A.^150^ This CLEC7A variant led to impaired Dectin-1 dimerization and surface expression. Functional analyses demonstrated that Dectin-1 stimulation promotes Tregs differentiation from naïve αβ and γδ T cells, even in the absence of transforming growth factor beta (TGF-*β*). Partial Dectin-1 deficiency exacerbated the Treg defect conferred by CTLA-4 haploinsufficiency, suggesting that Dectin-1 acts as a modifier gene, influencing the expressivity of CTLA4 variants and playing a role in maintaining immune homeostasis and tolerance.^150^ Moreover, recent studies have revealed that fungal components can upregulate a broader array of checkpoint and costimulatory molecules, including MHC-I, MHC-II, CD40, CD80, and CD86, particularly via co-engagement of Dectin-1 and TLR4 signaling in DCs.^151^ This upregulation supports enhanced antigen presentation and T cell priming, potentially boosting the efficacy of ICB therapies. Altogether, these data point to a fungi–CLR–checkpoint axis as a critical regulatory pathway in cancer immunology. Through their interaction with innate immune receptors, tumor-associated fungi can either promote immune tolerance or stimulate anti-tumor immunity depending on the context and immune cell type. These findings open new avenues for integrating mycobiome profiling and fungal-targeted therapies with checkpoint inhibition strategies in cancer treatment.

## The anti-tumor activity of YBG *β*-glucan

### *β*-glucan-induced reprogramming of innate immunity

Yeast-derived beta-glucan (YBG) enhances anti-tumor immunity by training innate immune cells, including macrophages, neutrophils, and DCs. Particularly, whole *β*-glucan particles (WGPs) promote macrophage reprogramming and antimetastatic activity in murine models of cancer metastasis.^152^ In lung metastasis models, WGPs induced a TI phenotype in interstitial macrophages, reducing metastatic burden and prolonging survival. This effect is driven by the metabolite sphingosine-1-phosphate (S1P), with its inhibition abrogating protection, highlighting the importance of the sphingolipid–mitochondrial axis in sustaining TI.^152^ WGPs also induce TI in human monocytes by activating multiple innate immune receptors (Dectin-1/complement receptor 3 (CR3), TLR4, and macrophage MR) and downstream signaling pathways, including Raf-1, SYK, and PI3K.^153^ This synergistic activation enhanced the secondary immune response to unrelated stimuli. In murine melanoma and bladder carcinoma models, *β*-glucan pre-treatment significantly reduced tumor growth.^153^ In PDA, YBG accumulates in the pancreas, triggering a CCR2-dependent recruitment of monocytes and macrophages exhibiting TI features.^154^ These cells become highly cytotoxic upon encountering tumor cells or tumor-derived signals. In murine models of PDA, YBG treatment significantly reduces tumor burden and extends survival, with even greater efficacy observed when combined with immunotherapeutic agents. Beyond macrophages, fungal-derived *β*-glucans also induce durable anti-tumor effects through transcriptional and epigenetic reprogramming of granulopoiesis and neutrophil function. This trained state skews neutrophils toward a tumoricidal phenotype, contributing to improved cancer control.^155^ Moreover, *β*-glucan-trained neutrophils from donor animals can be adoptively transferred to naïve recipients, where they inhibit tumor growth via a ROS-dependent mechanism. Furthermore, the tumor-suppressive programming induced by *β*-glucans during granulopoiesis can be transferred through bone marrow transplantation (BMT), emphasizing the systemic and durable nature of this reprogramming.^155^ These findings underscore the broad potential of *β*-glucans as immune-training agents that rewire the innate immune system for enhanced anti-tumor responses. A mechanistic overview of *β*-glucan-mediated immune modulation in cancer models is presented in Table 3.

**Table 3.** Mechanistic overview of *β*-glucan-mediated immune modulation in cancer models.

Mechanism	Effect	Details	Models/Systems	Model	References
**TI**	Anti-tumoral	Promotion of macrophage reprogramming; reduced metastasis, prolonged survival, S1P required for TI	Lung metastasis models	mouse	[152]
**TI**	Anti-tumoral	Activation of Dectin-1/CR3, TLR4, macrophage MR; and Raf-1, SYK, PI3K pathways; reduced tumor growth	Melanoma and bladder cell carcinoma	mouse + human primary monocytes + fungi	[153]
**TI**	Anti-tumoral	Recruitment of CCR2-dependent monocytes/macrophages with trained features, reduced tumor growth, prolonged survival	PDA	mouse + cell lines	[154]
**TI**	Anti-tumoral	Transcriptional and epigenetic reprogramming of granulopoiesis and neutrophil function → skewing neutrophils to a tumoricidal phenotype; *β*-glucan-trained neutrophils suppress tumors in naïve mice via ROS; *β*-glucan-induced anti-tumor programming transferable via BMT	Melanoma	mouse + cell lines	[155]
**Macrophage reprogramming**	Anti-tumoral	Conversion of naïve M0 macrophages + reprogramming M2-polarized macrophages and TAMs → tumoricidal M1 phenotype	Melanoma, lymphoma	mouse + cell lines	[156]
**Macrophage reprogramming**	Anti-tumoral	YBG–Ferumoxytol hybrid activates macrophages via Dectin-1 → strong M1 polarization via SYK/MAPK → ↑ TNF-*α*, IL-6, ROS → tumor cell apoptosis and arrest	Melanoma	mouse	[157]
**Initiation of adaptive immune responses**	Anti-tumoral	YBG-induced autophagy as a key mechanism in *β*-glucan-induced DCs activation	Melanoma, bladder cell carcinoma	mouse + tumor cell lines	[141]
**DCs modulation**	Anti-tumoral	Reversion of DCs’ dysfunction induced by tumor-derived factors → activation of CTLs + promotion of Th1-mediated immune responses	Lung cancer	mouse + cell lines	[158]
**MDSCs modulation**	Anti-tumoral	Induction of PMN-MDSC apoptosis → promotion of M-MDSC maturation into APCs → restoration of CD4^+^/CD8^+^ T cell activation	Lung cancer, mammary-cell carcinoma	mouse + human	[159]
**Activation of monocytes**	Anti-tumoral	Activation of monocytes → direct cytotoxicity against tumor cells + ↑ TNF-*α*, M-CSF, CCL2	lung metastatic melanoma	mouse + cell lines	[160]
**Complement activation (CR3/iC3b axis)**	Anti-tumoral	Oral YBG primes neutrophils via CR3 to kill iC3b-opsonized tumor cells; intravenous YBG forms immune complexes activating classical complement pathway→ augmentation of ADCP, ↑ ROS	Lymphoma, lung cancer	-	[144,145]
**ROS-mediated cytotoxicity**	Anti-tumoral	Increase of ROS in tumor cells → caspase 3/9 activation + cancer cell apoptosis	HeLa cervical cells	cell lines	[164]
**ROS-mediated cytotoxicity**	Anti-tumoral	Inhibition of autophagy by increasing lysosomal pH and blocking cathepsins B and D → mitochondrial dysfunction + ↑ ROS → caspase-8 activation + truncated BID translocation → cancer cells to apoptosis	mouse xenograft models, HCC	mouse + cell lines	[165]
**Complement overactivation (MBL–C3a axis)**	Pro-tumoral	Activation of lectin complement pathway; MBL/C3a signaling → PDA growth; deletion of MBL/C3/C3aR suppresses tumors	PDA models	mouse + cell lines	[56]

### Activation of immune signaling pathways to boost anti-tumor responses by YBG

YBG modulates both innate and adaptive immunity through diverse signaling cascades. One of the key mechanisms involves PRRs such as Dectin-1, initiating NF-κB, MAPK, and SYK pathways, leading to the production of pro-inflammatory cytokines and chemokines that amplify immune surveillance and cytotoxic responses. Oral administration of WGPs has been shown to delay tumor progression in various preclinical models by enhancing the functional activity of macrophages and NK cells.^156^ Notably, WGPs support macrophage polarization toward a tumoricidal M1 phenotype, not only by converting naïve M0 macrophages but also by reprogramming M2-polarized macrophages and TAMs, which are often co-opted by tumors to support immune evasion. In an innovative therapeutic approach, YBG has been conjugated with Ferumoxytol, an FDA-approved iron oxide nanoparticle, to form a nanocomposite with enhanced bioactivity.^157^ This hybrid material activates macrophages through Dectin-1 and induces robust M1 polarization via SYK and MAPK signaling, resulting in elevated TNF-*α*, IL-6, and ROS production. This pro-inflammatory shift facilitates tumor cell apoptosis and cell cycle arrest, as shown in models of melanoma and CRC.^157^ In addition to shaping macrophage and NK cell responses, YBG influences other critical aspects of tumor immunology. Ding et al showed that YBG-induced autophagy is a key mechanism in *β*-glucan-induced DCs activation, fostering their capacity to initiate effective adaptive immune responses.^141^ Moreover, YBG reverses DCs’ dysfunction induced by tumor-derived factors, restoring their ability to prime cytotoxic T lymphocytes (CTLs) and support Th1-mediated immune responses.^158^ This is particularly relevant given the role of Th1 cytokines like IFN-*γ* in promoting durable anti-tumor immunity. Another significant function of YBG is its impact on MDSCs, which play a pivotal role in tumor-induced immune suppression. YBG induces apoptosis in polymorphonuclear MDSCs (PMN-MDSCs), which suppress anti-tumor response and facilitates the differentiation of M-MDSCs into mature antigen-presenting cells (APCs) capable of activating CD4^+^ ​​​​and CD8^+^ T cells.^159^ By disrupting MDSC-mediated immunosuppression, YBG restores the effectiveness of both innate and adaptive immune responses, further enhancing its therapeutic value. Alexander et al., using a lung metastatic melanoma model, demonstrated that the anti-tumor effect of YGP can occur independently of adaptive immunity, instead relying on inflammatory monocytes.^160^ These YGP-activated monocytes exert direct cytotoxicity against tumor cells *in vitro*, and systemic YGP treatment increases the levels of inflammatory mediators such as TNF-*α*, M-CSF, and CCL2 in the lungs. Importantly, combination strategies involving YBG and ICIs or other immunotherapies have shown synergistic effects in preclinical models, indicating the potential of YBG as an adjunct agent in cancer immunotherapy.^161-163^ Collectively, these findings underscore the multifaceted role of YBG as a powerful modulator of the tumor immune microenvironment and a promising candidate for integrative cancer treatment strategies.

### YBG-induced complement activation and the role of ROS

Upon oral administration, fluorescein-labeled YBG is predominantly taken up by intestinal macrophages, which subsequently traffic the compound to central immune sites, including the spleen, lymph nodes, and bone marrow. Within the bone marrow microenvironment, degradation products of YBG interact specifically with CR3 expressed on neutrophils. This interaction initiates a signaling cascade that primes neutrophils to identify and eliminate tumor cells opsonized with the complement fragment iC3b, enhancing innate tumor surveillance and clearance.^145^ Conversely, intravenous delivery of YBG leads to its immediate association with circulating endogenous antibodies, forming immune complexes that activate the classical complement pathway. This activation results in the deposition of iC3b on tumor cells, which in turn facilitates engagement of immune effector cells via CR3 and the Fc region of the immunoglobulin G receptor IIa (FcγRIIA). The synergistic signaling through these receptors intensifies ROS production by neutrophils and augments macrophage-mediated antibody-dependent cellular phagocytosis (ADCP), collectively reinforcing anti-tumor immunity.^144^

In addition to its immunostimulatory effects, YBG-induced ROS play a critical role in mediating direct cytotoxicity toward tumor cells. Studies on *β*-glucan derived from *Pleurotus ostreatus* demonstrate that YBG elevates intracellular ROS levels, triggering apoptotic pathways in HeLa cervical cells.^164^ This oxidative stress is associated with marked nuclear condensation and fragmentation, hallmarks of apoptosis, and is accompanied by the activation of caspases 3 and 9 – key executioners of programmed cell death. In parallel, YBG exerts additional anti-tumor effects by interfering with cancer cell metabolic adaptation. Water-soluble yeast *β*-D-glucan (WSG) has been shown to function as a novel autophagy inhibitor, a process often hijacked by tumor cells to sustain growth under stress conditions.^165^ By increasing lysosomal pH and inhibiting the activity of cathepsins B and D, WSG disrupts autophagic degradation, leading to the accumulation of damaged mitochondria. This mitochondrial dysfunction results in a secondary surge in ROS, further amplifying oxidative stress. Elevated ROS levels not only directly impair tumor cell viability but also sensitize cancer cells to apoptosis by activating caspase-8 and mitochondrial translocation of truncated BH3 Interacting Domain Death Agonist (tBID), especially under nutrient-deprived conditions. *In vivo* experiments further support these observations as WSG administration significantly inhibited tumor growth without causing apparent toxicity in mouse xenograft models and chemically induced HCC (DEN/CCl₄) models.^165^ Thus, beyond enhancing immune-mediated tumor clearance, YBG exerts a dual anti-tumor effect by promoting ROS-dependent apoptosis in malignant cells, further underscoring its potential as a multifaceted therapeutic agent.

Beyond these mechanisms, recent studies have highlighted a pathogenic role of fungal-induced complement activation in PDA. Specifically, YBG can activate the lectin pathway of the complement system via binding to mannose-binding lectin (MBL). Ligation of MBL was shown to be essential for PDA progression: deletion of *Mbl* (encoding MBL) or *C3* in the extratumoral compartment, as well as knockdown of the C3a receptor (*C3aR*) in tumor cells, significantly suppressed tumor growth. These findings suggest that while YBG may harness complement signaling for therapeutic benefit, uncontrolled or context-specific fungal complement activation, particularly via the MBL–C3a axis, can paradoxically promote tumor development.^56^

## The fungal metabolome as a driver of tumor–immune interactions

Fungal metabolites are increasingly recognized as essential modulators of the TME, capable of influencing cancer progression and immune responses through diverse biochemical pathways. Among the most well-known of these metabolites are mycotoxins – toxic secondary metabolites produced by various fungal species – which have been extensively studied for their carcinogenic, genotoxic, and immunomodulatory properties. For instance, *Candida* species can produce acetaldehyde, a reactive metabolite implicated in DNA damage and local inflammation,^166^^,^^167^ as well as candidalysin, a cytolytic peptide toxin that disrupts epithelial barriers and activates immune signaling pathways.^168^^,^^169^ Other fungi, such as *Aspergillus* spp., may produce aflatoxins, well-known mycotoxins with genotoxic and carcinogenic potential.^170-174^ Apart from well known carcinogenic mycotoxins, such as candidalysin, aflatoxins, ochratoxins,^175-177^ fumonisins,^178-180^ and patulin^181^^,^^182^ that can damage DNA or induce chronic inflammation and immune dysregulation, fungi produce a variety of other bioactive metabolites that can modulate the TME and immune cell function, thereby influencing the efficacy of ICIs. These metabolic interactions may contribute to either immunosuppressive or immunostimulatory microenvironments, thereby affecting tumor immune surveillance and responsiveness to ICIs. For instance, fungi produce eicosanoids, including PGE_2,_ prostaglandin D2 (PDE_2_), and F2α (PDF_2α_),^183^ which can influence both innate and adaptive immune responses.^184-186^ PGE_2_ plays a key role in tumor immune evasion by suppressing innate and adaptive immune responses, including T cell activity, thereby reducing the effectiveness of immunotherapies.^185^^,^^187^^,^^188^ Targeting the PGE_2_–EP4 signaling axis can help restore anti-tumor immunity. The selective EP4 antagonist HTL0039732 reverses PGE_2_-driven M2-like macrophage polarization and enhances immune responses, and preclinical studies demonstrate its synergy with PD-1/PD-L1 blockade.^189^ PGD_2_ creates an autocrine loop that maintains an immunosuppressive, protumoral phenotype. Through DP1/DP2 receptors, PGD_2_ inhibits CD8^+^ T-cell activity; blocking PGD_2_ activity enhances T-cell responses and sensitizes tumors to anti-PD-1 therapy.^190^

On the other hand, fungal-derived metabolites such as chromone,^191-193^ ketones (e.g., Brocaeloid D from *Microdochium majus,*^194^ depsidones and diaryl ethers from potato fungus *Boeremia exigua*^195^), sesquiterpenoids,^196^ and cytochalasins^197^ have demonstrated significant anticancer properties through mechanisms involving apoptosis induction, cell cycle arrest, and immune lmodulation. In addition to these compounds, several fungal species have been identified as sources of potent anti-tumor agents with potential therapeutic applications. He et al. reported that endophytic fungal secondary metabolites from *Ginkgo biloba* leaves significantly reduced tumor formation in HeLa-implanted mice by promoting apoptosis and inhibiting proliferation of HeLa cells.^198^ Similarly, extracellular polysaccharides from *Schizophyllum radiatum* showed anti-tumor activity.^199^ Schisandra polysaccharide, a product of *Schisandra chinensis* fermentation, exhibited strong immunomodulatory effects *in vivo* and enhanced anti-tumor properties.^200^^,^^201^ FIP-nha, a 114-amino-acid protein isolated from *Nectria haematococca*, suppressed lung cancer cell growth *in vivo* by inhibiting the PI3K/Akt pathway.^202^ Likewise, colletofragarone A2, derived from *Colletotrichum* spp., reduced mutant p53 levels and inhibited tumor progression.^203^ Furthermore, a novel L-asparaginase produced by *Yarrowia lipolytica* was shown to suppress the proliferation and migration of both lung and breast cancer cells.^204^

**Figure 3.**
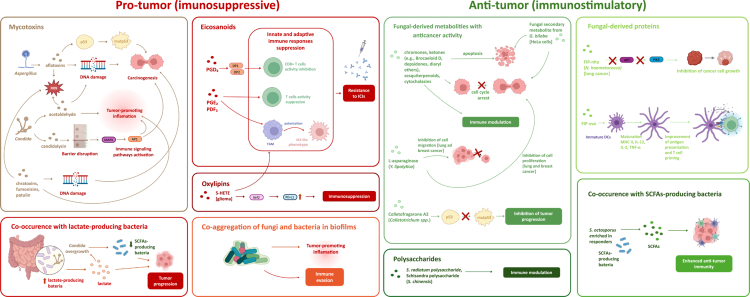
Dual roles of fungal metabolites in cancer highlighting both pro-tumor (immunosuppressive) and anti-tumor (immunostimulatory) mechanisms. Fungi contribute to both pro-tumor (red, left) and anti-tumor (green, right) mechanisms. Pro-tumor effects include DNA damage, barrier disruption, immune suppression, resistance to ICIs, and tumor-promoting interactions with bacteria. In contrast, fungal-derived metabolites, proteins, and polysaccharides can exert anticancer activity by inducing apoptosis, inhibiting proliferation, modulating immunity, and enhancing anti-tumor responses, including through co-occurrence with SCFA-producing bacteria.

Oxylipins, a class of oxygenated fatty acid derivatives, another fungal metabolites, are known to influence macrophage polarization and PD‑L1 expression in the TME. For instance, in glioma, oxylipin 5-hydroxyeicosatetraenoic acid (5-HETE) promoted M2-like macrophage polarization and PD‑L1 upregulation via the nuclear factor erythroid 2-related factor 2 (Nrf2) pathway, leading to immunosuppressive TME.^205^ In fungal infection models, fungal oxylipins have been reported to modulate macrophage polarization and downstream immune checkpoint signaling.^206^^,^^207^ Other fungal metabolites, including fermentation products such as ethanol, organic acids, and fungal immunomodulatory proteins, profoundly affect APC phenotype and function. For example, fungal immunomodulatory proteins like FIP‑vvo enhance DCs maturation (elevated MHC II, IL‑12, IL‑2, TNF‑α), improving antigen presentation and T cell priming.^208^ These findings underscore the growing recognition of the fungal metabolome as a source of diverse compounds capable of modulating cancer biology and immune responses. As such, exploring the role of fungal metabolites in shaping the immunological landscape of cancer may reveal new avenues for enhancing the efficacy of immunotherapies. Immunomodulatory and oncogenic effects of fungal metabolites and mycotoxins are summarized in Table 4 and Figure 3.

**Table 4.** Immunomodulatory and oncogenic effects of fungal metabolites and mycotoxins.

Effect	Representative Metabolites/Compounds	Mechanism	Model	References
**Carcinogenic metabolite**	acetaldehyde	epithelial dysplasia, DNA adduct formation, inhibition of DNA repair, oxidative stress, local inflammation, promotion of mutagenesis and carcinogenesis	human + fungi	[166,167]
**Carcinogenic mycotoxins**	candidalysin	epithelial barrier disruption, induction of proinflammatory cytokines, activation of MAPK/AP-1 pathway, promotion of tissue damage and inflammation-mediated carcinogenesis	cell lines + organoid	[168,169]
**Carcinogenic mycotoxins**	aflatoxins	genotoxicity, immunosuppression, DNA adduct formation, oxidative stress, p53 mutation, general tumor promotion	-	[170–172]
**Carcinogenic mycotoxins**	ochratoxins	genotoxicity, nephrotoxicity, neurotoxicity, embryotoxicity, teratogenicity, carcinogenicity, inhibition of protein synthesis, oxidative damage, DNA strand breaks	-	[175–177]
**Carcinogenic mycotoxins**	fumonisins	hepatotoxicity, neurotoxicity, carcinogenicity, disruption of sphingolipid metabolism (inhibits ceramide synthase)	pig	[178–180]
**Carcinogenic mycotoxins**	patulin	immunotoxicity, genotoxicity, ROS generation, mitochondrial damage, DNA damage	mouse + cell lines	[181,182]
**Immunosuppression**	PGE_2_, PGD_2_, PDF_2α_	inhibits T cells, promotes M2 macrophages	mouse + human	[183–190]
**Immunosuppression**	PGD_2_	inhibits CD8^+^ T-cell activity through DP1/DP2 receptors; blocking PGD2 activity enhances T-cell responses and synergizes with anti-PD-1 therapy	mouse + cell lines	[190]
**Anti-cancer secondary metabolites**	Chromones, ketones, sesquiterpenoids, cytochalasins	promotion of apoptosis, cell cycle arrest, immune modulation	fungi	[191–197]
**Anti-cancer secondary metabolites**	endophytic fungal secondary metabolites from *G. biloba* leaves	promotion of apoptosis and inhibition of the proliferation of HeLa cells	mouse	[198]
**Anti-tumor polysaccharides**	*S. radiatum* extracellular polysaccharides, Schisandra polysaccharide	immune activation, enhances immune responses	mouse + fungi	[199–201]
**Anti-tumor fungal proteins**	FIP-nha (from *N. haematococca*)	suppresses the PI3K/Akt pathway in lung cancer model	mouse	[202]
**Anti-tumor**	Colletofragarone A2 (from *Colletotrichum* spp.)	p53-dependent tumor suppression	fungi	[203]
**Anti-tumor enzymes**	L-asparaginase (from *Y. lipolytica*)	suppresses the proliferation of lung and breast cancer cells	cell lines	[204]
**Immunosuppression**	Oxylipins (e.g., 5-HETE)	promotes PD-L1 upregulation + M2-like macrophage polarization in glioma via the Nrf2 pathway	human + mouse	[205–207]
**Anti-tumor protein**	FIP-vvo	↑ DC maturation (↑ MHC II, IL-12, TNF-*α*) → improved antigen presentation and T cell priming	mouse	[208]

## Conclusions

The gut mycobiome is increasingly recognized as an important, though often neglected, component of the tumor–microbiota–host axis. While bacterial communities have long been the focus of immuno-oncology research, recent findings suggest that fungi also play a critical role in shaping immune responses and influencing cancer progression and treatment efficacy. The mycobiome does not act in isolation; it interacts intricately with bacterial consortia and the host immune system, contributing to a dynamic ecological and immunological environment that can either support or hinder anti-tumor immunity. These interactions are not merely theoretical; they carry real clinical implications. Fungal genera such as *Candida* and *Malassezia* have been linked to key immunomodulatory pathways, potentially affecting ICB. Mechanistically, fungi engage the host immune system through conserved cell wall components such as *β*-glucans, mannans, and chitin, which are recognized by PRRs including Dectin-1, TLRs, and NLRs. These interactions can shape innate and adaptive responses, influencing T cell polarization and cytokine production. In addition, fungal metabolism generates a range of bioactive secondary metabolites, which can further modulate the TME and immune tone. Such metabolites may enhance inflammation, promote immune suppression, or interfere with antigen presentation. As such, comprehensive immunotherapeutic strategies must begin to account for the fungal dimension of the microbiome to improve patient stratification, predict outcomes, and guide interventions. Integrating fungal data into clinical oncology and microbiome-based therapeutics will require a paradigm shift. A mechanistic and translational understanding of fungi–host–tumor interactions is essential to fully exploit the therapeutic potential of the microbiome.

## Abbreviations

**ADCP**: antibody-dependent cellular phagocytosis
**AML**: acute myeloid leukemia
**APCs**: antigen-presenting cells
**AUC**: area under the curve
BMT: bone marrow transplantation
**CARD9**: caspase recruitment domain-containing protein 9
**CAR**: chimeric antigen receptor
**CBM complex**: CARD9–BCL10–MALT1 signaling complex
**CLRs**: C-type lectin receptors
**CRC**: colorectal cancer
**CRD**: carbohydrate recognition domain
**CR3**: complement receptor 3
**CTLA-4**: cytotoxic T-lymphocyte antigen-4
**CTLs**: cytotoxic T lymphocytes
**DCs**: dendritic cells
**DC-SIGN**: dendritic cell-specific intercellular adhesion molecule-3-grabbing non-integrin
**ERK**: extracellular signal-regulated kinase
**ERMAP**: erythroid membrane-associated protein
**FcRγ**: Fc receptor γ-chain
**FcγRIIA**: Fc region of the immunoglobulin G receptor IIa
**FMT**: fecal microbiota transplantation
**GOSs**: galacto-oligosaccharides
**HCC**: hepatocellular carcinoma
**ICAM3**: intercellular adhesion molecule 3
**ICB**: immune checkpoint blockade
**ICI**/**ICIs**: immune checkpoint inhibitor(s)
**IFN-γ**: interferon gamma
**IL**: interleukin
**ILC3s**: group 3 innate lymphoid cells
**IRF4**: interferon regulatory factor 4
**iRAEs**: immune-related adverse events
**ITAM**: immunoreceptor tyrosine-based activation motif
**JAK**: Janus kinase
**mABs**: antagonistic monoclonal antibodies
**MAPK**: mitogen-activated protein kinase
**MALT1**: mucosa-associated lymphoid tissue lymphoma translocation protein 1
**MBL**: mannose-binding lectin
**MDSCs**: myeloid-derived suppressor cells
**Mincle**: macrophage-inducible C-type lectin (CLEC4E)
**MMR**: macrophage mannose receptor
**MR**: mannose receptor
**M-MDSCs**: monocytic MDSCs
**NIK**: NF-κB-inducing kinase
**NK cells**: natural killer cells
**NLRs**: NOD-like receptors
**NSCLC**: non-small cell lung cancer
**OSCC**: oral squamous cell carcinoma
**PAMPs**: pathogen-associated molecular patterns
**PDA**: pancreatic ductal adenocarcinoma
**PD**: programmed death
**PD-L1/PD-L2**: programmed death ligand-1/ligand-2
**PDE_2_**: prostaglandin D2
**PDF_2α_**: prostaglandin F2α
**PGE₂**: prostaglandin E₂
**PGF₂**: prostaglandin F₂
**PKC-δ**: protein kinase Cδ
**PMN-MDSCs**: polymorphonuclear MDSCs
**PRRs**: pattern recognition receptors
**RCC**: renal cell carcinoma
**RIG-I/RLRs**: retinoic acid-inducible gene I (RIG-I)-like receptors
**ROS**: reactive oxygen species
**SAPs**: secreted aspartyl proteases
**SCFA**: short-chain fatty acid
**SYK**: spleen tyrosine kinase
**S1P**: sphingosine-1-phosphate
**TAMs**: tumor-associated macrophages
**tBID**: truncated BH3 Interacting Domain Death Agonist
**TCR**: T cell receptor
**TDB**: Trehalose dibehenate
**TDM**: trehalose-6,6′-dimycolate
**TGF-β**: transforming growth factor beta
**Th**: T helper
**TI**: trained immunity
**TLR**: Toll-like receptor
**TME**: tumor microenvironment
**TNF-α**: tumor necrosis factor alpha
**WGPs**: whole β-glucan particles
**WSG**: Water-soluble yeast β-D-glucan
**YBG**: yeast-derived β-glucan
**5-HETE**: 5-hydroxyeicosatetraenoic acid
